# Somatic Mosaic *AXIN2*‐associated Colonic Polyposis Predominantly Involving the Proximal Colon: A Case Report

**DOI:** 10.1002/deo2.70409

**Published:** 2026-08-24

**Authors:** Takashi Murakami, Masami Arai, Hidetaka Eguchi, Yuka Hirasawa, Wataru Hata, Yudai Otsuki, Tomonori Yamauchi, Eiji Kamba, Takashi Yao, Akihito Nagahara

**Affiliations:** ^1^ Department of Gastroenterology Juntendo University Faculty of Medicine Tokyo Japan; ^2^ Department of Clinical Genetics Graduate School of Medicine Juntendo University Tokyo Japan; ^3^ Diagnostics and Therapeutics of Intractable Diseases Intractable Disease Research Center Graduate School of Medicine Juntendo University Tokyo Japan; ^4^ Department of Human Pathology Juntendo University Graduate School of Medicine Tokyo Japan; ^5^ Department of Pathophysiological Research and Therapeutics for Gastrointestinal Disease Juntendo University Faculty of Medicine Tokyo Japan

**Keywords:** AXIN2, colonic polyposis, endoscopic submucosal dissection, somatic mosaicism, Wnt signaling pathway

## Abstract

Colonic adenomatous polyposis is most commonly caused by germline pathogenic variants in the *APC* gene; however, recent genetic studies have identified patients without *APC* mutations. *AXIN2*‐associated colonic polyposis is a rare condition related to dysregulation of the Wnt/β‐catenin signaling pathway. Most reported cases have involved germline *AXIN2* variants, whereas somatic mosaic *AXIN2* alterations remain poorly documented. We report a rare case of somatic mosaic *AXIN2*‐associated colonic polyposis predominantly involving the proximal colon. A 77‐year‐old asymptomatic man was referred for colonoscopic surveillance after previous polypectomy. Colonoscopy revealed multiple adenomatous polyps from the cecum to the proximal ascending colon, with some lesions up to 10 mm in size, while only a few small polyps were observed in the transverse and descending colon and none in the sigmoid colon or rectum. The patient underwent intensive endoscopic management, including endoscopic submucosal dissection for extensive adenomatous mucosa in the cecum, followed by repeated endoscopic resections and close surveillance. Histopathological examination confirmed low‐grade tubular adenomas. Because of multiple adenomas, genetic testing was performed after genetic counseling. No pathogenic variants were detected in peripheral blood. Analysis of independently resected colonic polyps consistently identified the same heterozygous frameshift mutation in *AXIN2* with loss of heterozygosity, whereas no *AXIN2* abnormalities were detected in normal colonic mucosa, leading to a definitive diagnosis of somatic mosaic *AXIN2*‐associated colonic polyposis. This case highlights a previously unreported mechanism of *AXIN2*‐driven colonic polyposis through somatic mosaicism and suggests that intensive endoscopic treatment with close surveillance may be an effective management strategy in selected patients.

## 1 Introduction

Colonic adenomatous polyposis is most commonly represented by familial adenomatous polyposis (FAP), a hereditary disorder characterized by the development of numerous colorectal adenomas and a markedly increased risk of colorectal cancer [1]. Classical FAP is caused by germline pathogenic variants in the *APC* gene, a central regulator of the Wnt/β‐catenin signaling pathway [1, 2].

However, advances in genetic testing have shown that a subset of patients with adenomatous polyposis do not harbor *APC* pathogenic variants, and comprehensive multigene approaches are increasingly recommended for accurate molecular diagnosis [3]. Among the non‐*APC* genes, *AXIN2* has emerged as a rare but clinically important cause of adenomatous polyposis. *AXIN2* encodes a scaffold protein within the β‐catenin destruction complex and functions as a negative regulator of Wnt signaling [2]. Germline *AXIN2* pathogenic variants have been associated with attenuated colonic polyposis and colorectal cancer susceptibility, often with variable penetrance and occasional extracolonic features such as tooth agenesis [4, 5, 6].

In contrast, while somatic mosaicism of *APC* is a well‐recognized mechanism in apparently sporadic polyposis, somatic mosaic *AXIN2* alterations have not been well documented in human polyposis. Here, we report a rare case of *AXIN2*‐associated colonic polyposis caused by somatic mosaicism, predominantly involving the proximal colon and successfully managed with intensive endoscopic treatment.

## 2 Case Presentation

A 77‐year‐old asymptomatic man was referred for colonoscopic surveillance following previous polypectomy for colonic adenomas. At the age of 72, he had undergone endoscopic polypectomy for several adenomas in the sigmoid colon at another institution. His medical history included hypertension and cerebral infarction. There was no known family history of colorectal cancer or polyposis syndromes.

**FIGURE 1:**
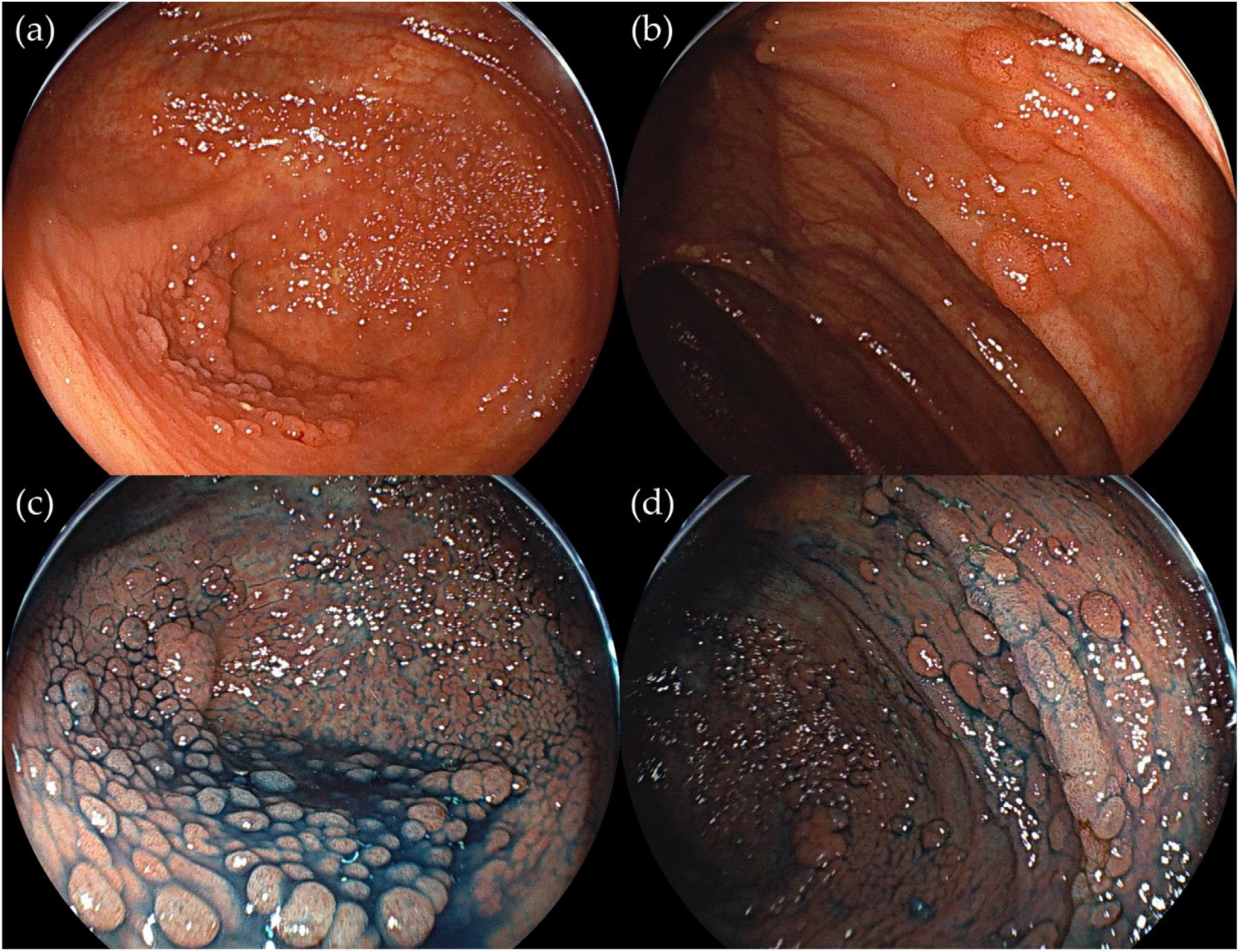
Endoscopic findings of multiple colonic polyps. (a) White‐light endoscopic view of the cecum showing numerous polyps densely clustered in the cecal mucosa. (b) White‐light endoscopic view of the proximal ascending colon demonstrating multiple polyps scattered throughout the mucosa. (c) Indigo carmine chromoendoscopy of the cecum highlighting the clustered polypoid lesions. (d) Indigo carmine chromoendoscopy of the proximal ascending colon showing clear delineation of the scattered polyp surfaces.

**FIGURE 2:**
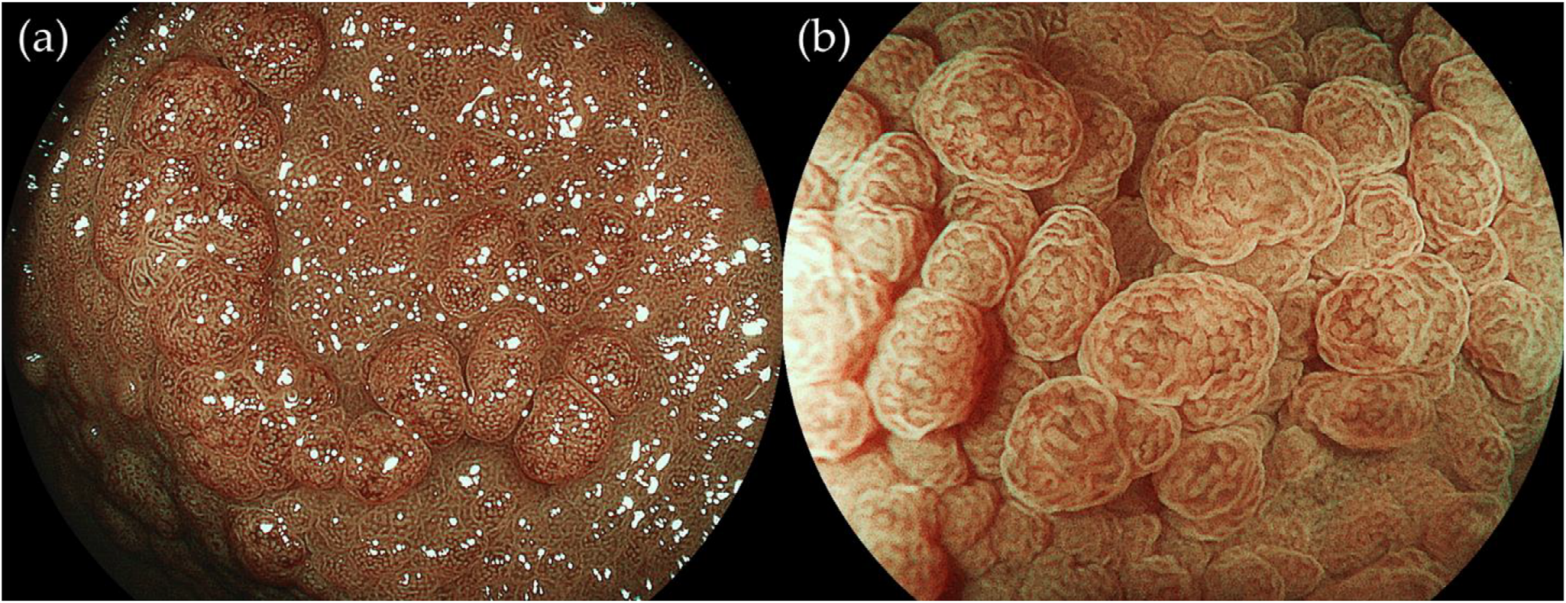
Magnifying blue laser imaging (BLI) of multiple colonic polyps. (a) BLI with low magnification showing regular microvascular and surface patterns. (b) BLI with medium magnification demonstrating regular vessel and surface structures consistent with adenomas (Japan NBI Expert Team classification, type 2A).

Colonoscopy revealed more than 200 adenomatous polyps predominantly involving the cecum and proximal ascending colon, with some lesions measuring up to 10 mm in diameter (Figure 1). Most polyps were hemispherical, while several lesions were fused and flattened. Magnifying blue laser imaging using a Fujifilm endoscopy system (Tokyo, Japan) showed regular microvascular and surface patterns, consistent with type 2A according to the Japan NBI Expert Team (JNET) classification (Figure 2). Only a few small polyps (<5 mm) were identified in the transverse and descending colon, and no polyps were detected in the sigmoid colon or rectum (Figure S1).

**FIGURE 3:**
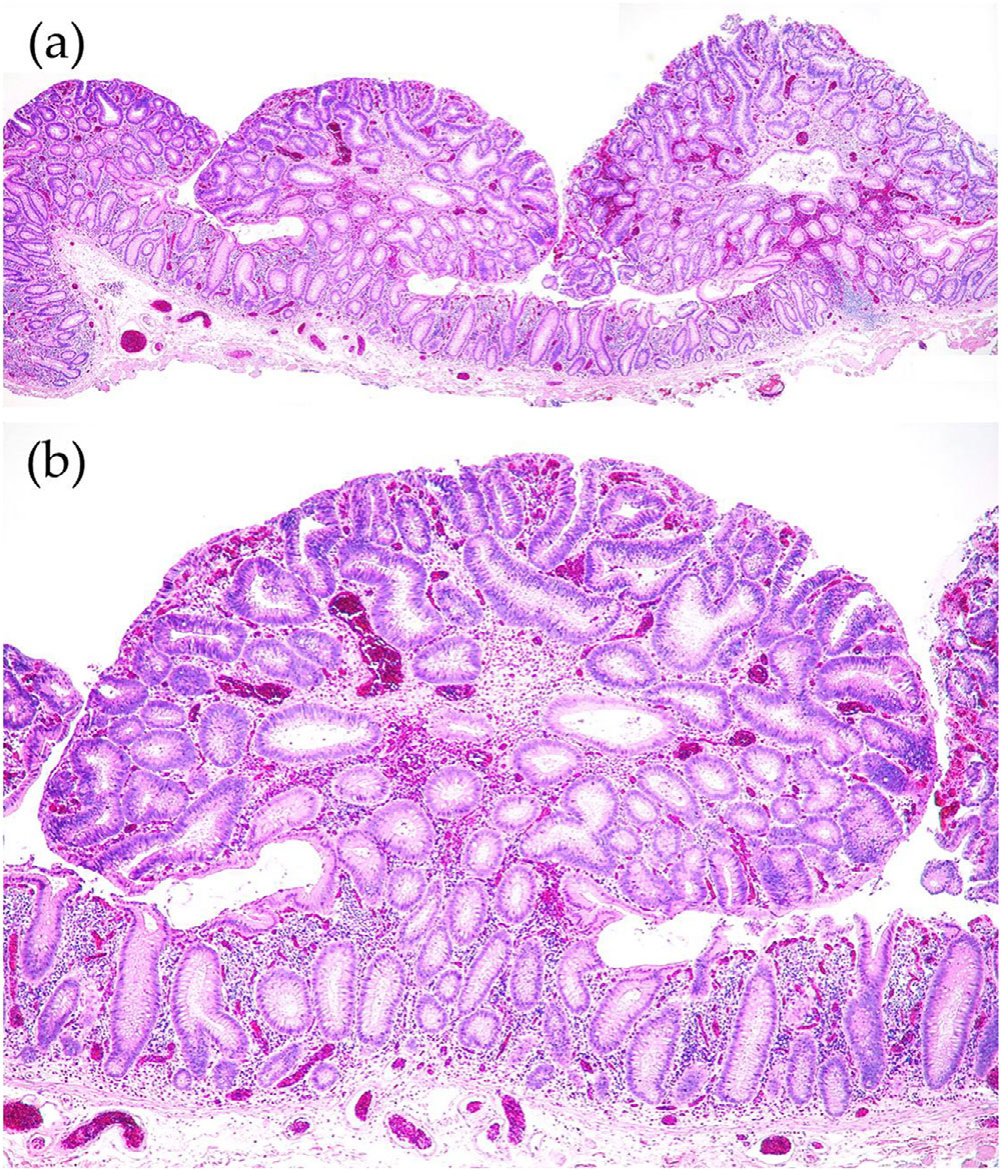
Histopathological findings of the resected specimen (Hematoxylin and eosin staining). (a) Low‐power view showing a cluster of multiple small adenomatous polyps composed of crowded tubular glands. (b) Medium‐power view demonstrating adenomatous glands with nuclear pseudostratification and mild cytological atypia, consistent with low‐grade tubular adenoma.

The lesions were clinically diagnosed as adenomas. Endoscopic submucosal dissection (ESD) was performed to resect the extensive adenomatous mucosa in the cecum. Histopathological examination confirmed low‐grade tubular adenomas without high‐grade dysplasia or invasive carcinoma (Figure 3). No hyperplastic polyps or serrated lesions were identified in any of the resected specimens. Given the localized distribution of the adenomatous lesions, a staged endoscopic treatment strategy was adopted to achieve complete eradication while preserving colonic function. A second ESD was performed three months later to remove the remaining adenomatous mucosa in the cecum. Residual polyps were subsequently treated with cold snare polypectomy at 6‐month intervals. After five planned endoscopic treatment sessions, follow‐up colonoscopy demonstrated post‐ESD scar formation with no residual or recurrent adenomas in the cecum (Figure S2).

**FIGURE 4:**
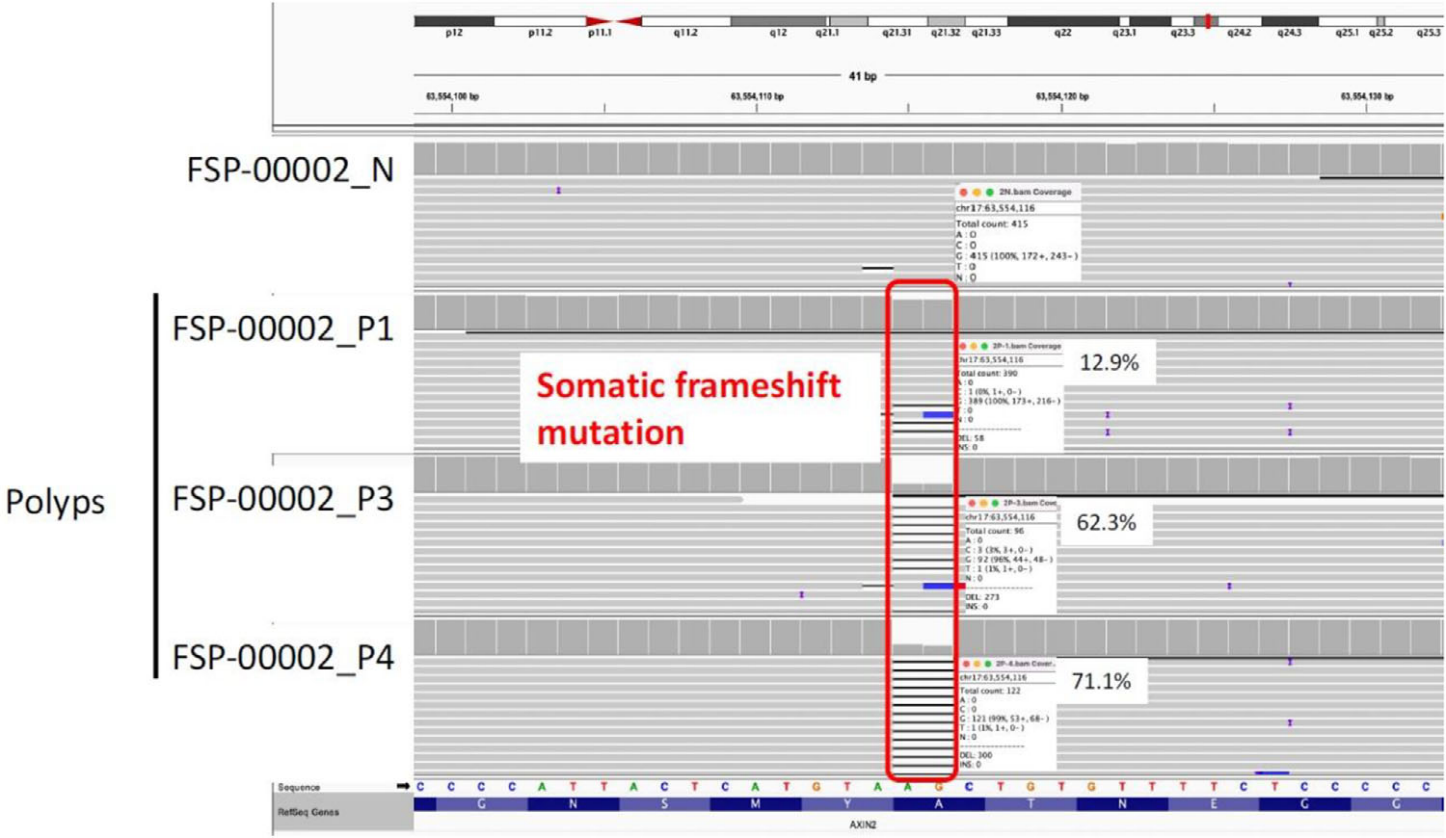
Molecular evidence supporting somatic mosaic *AXIN2*‐associated colonic polyposis. Detection of the *AXIN2* c.623_624delCT Mutation. Representative Integrative Genomics Viewer (IGV) images demonstrating the *AXIN2* frameshift mutation (c.623_624delCT, p.Ala208fs) in three independent adenomas (P1, P3, and P4), whereas no corresponding mutation was detected in matched normal colonic mucosa. Variant allele frequencies (VAFs) were 12.9%, 62.3%, and 71.1% in P1, P3, and P4, respectively.

Because of multiple colorectal adenomas, the patient was referred for genetic counseling for suspected FAP, despite the absence of a family history. After obtaining written informed consent, targeted next‐generation sequencing was performed using peripheral blood DNA and a hereditary colorectal cancer multigene panel that included *AXIN2* and other established polyposis‐ and colorectal cancer–associated genes (Table S1). No pathogenic variants were detected in peripheral blood, including *APC*, *MUTYH*, *POLE*, and *POLD1*. Next, DNA extracted from three independent colonic polyps, all confirmed as low‐grade tubular adenomas, together with three samples of endoscopically normal colonic mucosa (one from the cecum and two from the ascending colon within the polyposis‐affected region) was analyzed using the same gene panel. No pathogenic *APC* variants were identified. In contrast, a heterozygous frameshift variant in the *AXIN2* gene (c.623_624delCT), accompanied by loss of heterozygosity, was identified in all three adenomas (Figure 4 and Figure S3). No *AXIN2* abnormalities were detected in any of the normal mucosal samples. The patient was definitively diagnosed with somatic mosaic *AXIN2*‐associated colonic polyposis.

## 3 Discussion

*AXIN2* is a key negative regulator of the Wnt/β‐catenin signaling pathway through its role as a scaffold protein in the β‐catenin destruction complex [2]. Germline pathogenic variants in *AXIN2* are a rare cause of hereditary colorectal adenomatous polyposis, typically presenting with an attenuated phenotype and variable clinical expression [4, 5, 6]. However, the clinical significance of somatic mosaic *AXIN2* alterations in human polyposis has remained unclear.

In classical FAP, a germline *APC* pathogenic variant is present in all cells from conception, explaining the early onset, diffuse polyposis, frequent extracolonic manifestations, and strong family history [1, 7]. In contrast, somatic mosaicism of *APC* is a well‐established mechanism underlying a subset of apparently sporadic FAP or attenuated FAP cases, particularly when pathogenic variants are not detected in peripheral blood [8]. This concept provides a framework for interpreting the present case. Our findings suggest that somatic mosaicism can also affect *AXIN2*, producing a phenotype resembling hereditary polyposis. To the best of our knowledge, this is the first report of *AXIN2*‐associated colonic polyposis caused by somatic mosaicism rather than a germline variant.

A key observation was the absence of pathogenic variants in peripheral blood despite clinically significant adenomatous polyposis. This scenario is increasingly encountered, and recent studies emphasize the diagnostic value of multigene testing and integrated interpretation of germline and tissue‐based analyses in *APC*‐negative polyposis [3]. In the present case, analysis of multiple independent colonic polyps consistently identified the same *AXIN2* frameshift mutation together with loss of heterozygosity, whereas no *AXIN2* abnormalities were detected in normal colonic mucosa. This pattern strongly supports a postzygotic origin and a plausible “two‐hit” mechanism for tumorigenesis in the affected colonic mucosa, consistent with the established role of Wnt pathway dysregulation in adenoma development [2].

Our patient showed later onset and a limited adenoma burden predominantly involving the proximal colon, with no rectal involvement. In addition, no extracolonic manifestations typically associated with *APC*‐driven FAP, such as duodenal adenomas or desmoid tumors, were observed. The absence of dental abnormalities or ectodermal findings is not inconsistent with somatic mosaicism, in which the pathogenic variant may be confined to colonic tissues [4, 5, 6]. These findings suggest that the phenotypic spectrum of *AXIN2*‐associated polyposis may be broader than previously recognized.

From a therapeutic standpoint, prophylactic colectomy remains the standard approach for classical FAP because of the near certainty of colorectal cancer development [7]. In the present case, however, intensive endoscopic management, including ESD for extensive adenomatous mucosa, achieved effective disease control while preserving colonic function. This strategy involved repeated endoscopic resection of visible adenomas combined with close surveillance. Three years after ESD, the patient remains in good general condition without evidence of recurrence or progression. This favorable course suggests that when a causative alteration is somatic mosaic rather than germline, a colon‐preserving strategy may be feasible in carefully selected patients. Such patients may include those with polyposis confined to a limited colonic segment, without advanced neoplasia, and in whom complete endoscopic control can be achieved through repeated treatment and meticulous surveillance.

Finally, this case highlights the value of comprehensive genetic testing using peripheral blood and lesion tissue. Although the *AXIN2* alteration was not germline and therefore not directly transmissible, accurate molecular characterization remains clinically meaningful for patient education and genetic counseling. More importantly, understanding the natural history of rare mosaic polyposis is essential for optimizing individualized management and avoiding unnecessary aggressive interventions. Accumulation of additional cases will be required to clarify the clinical course and to establish evidence‐based surveillance and management strategies for this emerging entity.

## Author Contributions

All authors contributed to this work. **Takashi Murakami** and **Masami Arai** contributed to the conception and design of the study. **Takashi Murakami**, **Yuka Hirasawa**, **Wataru Hata**, **Yudai Otsuki**, **Tomonori Yamauchi**, and **Eiji Kamba** performed the endoscopic procedures and collected and analyzed the clinical data. **Takashi Yao** performed the pathological evaluation. **Masami Arai** provided genetic counseling to the patient. **Masami Arai** and **Hidetaka Eguchi** contributed to the genetic analysis and interpretation. **Takashi Murakami** drafted the manuscript. **Masami Arai**, **Hidetaka Eguchi**, and **Akihito Nagahara** critically revised the manuscript. All authors read and approved the final version of the manuscript.

## Funding

The authors have nothing to report.

## Ethics Statement

This case report was conducted in accordance with the principles of the Declaration of Helsinki. Institutional review board approval was not required for this single case report in accordance with our institutional policy.

## Consent

Written informed consent was obtained from the patient for genetic testing and for the publication of this case report and any accompanying images.

## Conflicts of Interest

The authors declare no conflicts of interest.

## Supporting information




**Supporting File1**: deo270409‐sup‐0001‐SuppMat.zip
