# Supplementary material for: Somatic Mosaic AXIN2‐associated Colonic Polyposis Predominantly Involving the Proximal Colon: A Case Report
Source: DEN Open. 2026 Aug 24;7(1):e70409. doi: 10.1002/deo2.70409 (PMC13504014; doi:10.1002/deo2.70409)
Supplement: Supplementary file 1 [file DEO2-7-e70409-s001.zip › Supplementary Table 1. Gene Panel (Murakami, et al).docx]

# Supplementary Table 1

**Genes included in the hereditary colorectal cancer multigene panel**

APC, MUTYH, POLD1, POLE, NTHL1, SCG5, GREM1, AXIN2, MLH1, MSH2, MSH6, PMS2, EPCAM, MCM9, PMS1, MSH3, MLH3, SMAD4, BMPR1A, PTEN, STK11, SDHB, SDHD, SMAD9, ENG, CDH1, TP53, RNF43, BRCA1, BRCA2, BARD1, BRIP1, BUB1, BUB1B, BUB3, FANCC, FANCE, FAN1, ATM, RAD52, CHEK2, XRCC4, PIK3CA, AKT1, CDKN1B, TGFBR2, SMARCA4, MBD4, RPS20, GALNT12, LRP6, RBL1, REV3L, CNTN6, XAF1, EPHX1, NFKBIZ, POLQ, UIMC1, TDRD3, and MYH11.

**Footnote.** Targeted next-generation sequencing was performed using a hereditary colorectal cancer multigene panel comprising the above 61 genes.
